# Electromagnetic and Dynamic Mechanical Properties of Epoxy and Vinylester-Based Composites Filled with Graphene Nanoplatelets

**DOI:** 10.3390/polym8080272

**Published:** 2016-07-28

**Authors:** Fabrizio Marra, Alessandro Giuseppe D’Aloia, Alessio Tamburrano, Isabel Maria Ochando, Giovanni De Bellis, Gary Ellis, Maria Sabrina Sarto

**Affiliations:** 1 Department of Astronautics, Electrical and Energy Engineering, Sapienza University of Rome, 00184 Rome, Italy; fabrizio.marra@uniroma1.it (F.M.); alessandrogiuseppe.daloia@uniroma1.it (A.G.D.); alessio.tamburrano@uniroma1.it (A.T.); giovanni.debellis@uniroma1.it (G.D.B.); 2 Research Center for Nanotechnology applied to Engineering, Sapienza University of Rome, 00184 Rome, Italy; 3 Institute of Polymer Science and Technology ICTP-CSIC, Consejo Superior de Investigaciones Científicas CSIC, 28006 Madrid, Spain; iochando@ictp.csic.es (I.M.O.); gary@ictp.csic.es (G.E.)

**Keywords:** polymer composites, graphene nanoplatelets, radar absorbing materials, electromagnetic properties, DC electrical conductivity, percolation threshold, mechanical properties, effective dielectric permittivity, DMTA

## Abstract

Development of epoxy or epoxy-based vinyl ester composites with improved mechanical and electromagnetic properties, filled with carbon-based nanomaterials, is of crucial interest for use in aerospace applications as radar absorbing materials at radio frequency. Numerous studies have highlighted the fact that the effective functional properties of this class of polymer composites are strongly dependent on the production process, which affects the dispersion of the nanofiller in the polymer matrix and the formation of micro-sized aggregations, degrading the final properties of the composite. The assessment of the presence of nanofiller aggregation in a composite through microscopy investigations is quite inefficient in the case of large scale applications, and in general provides local information about the aggregation state of the nanofiller rather than an effective representation of the degradation of the functional properties of the composite due to the presence of the aggregates. In this paper, we investigate the mechanical, electrical, and electromagnetic properties of thermosetting polymer composites filled with graphene nanoplatelets (GNPs). Moreover, we propose a novel approach based on measurements of the dielectric permittivity of the composite in the 8–12 GHz range in order to assess the presence of nanofiller aggregates and to estimate their average size and dimensions.

## 1. Introduction

During the last two decades, the use of carbon-based nanofillers in polymeric matrices has been widely investigated within the scope of producing multifunctional polymer composites with enhanced properties [1,2,3,4]. In particular, for aeronautical and electromagnetic compatibility applications, the development of thermosetting composites with improved electrical and mechanical properties is a challenge in the work towards the production of lightweight structural radar absorbing materials (RAMs) or electromagnetic (EM) shields against high intensity radiated field (HIRF) or lightning indirect effects.

Carbon nanotubes (CNTs), graphene oxide (GO), multilayer graphene microsheets (MLGs), and graphene nanoplatelets (GNPs) offer a combination of excellent thermal conductivity, mechanical, electrical, and electronic transport properties [5,6]. The EM shielding properties of carbon-based composites filled with different types of carbonaceous nanofillers have been investigated in the literature, considering a range of different polymer systems [7,8,9,10,11,12,13,14,15,16,17,18,19]. Recent studies have demonstrated that for applications such as RAM, polymer composites filled with 2D-carbon nanomaterials—like MLGs or GNPs—can be highly competitive with respect to CNT-filled composites and offer a promising alternative to the use of multiphase systems [13,14,15]. 

MLGs are stacks of graphene sheets obtained by the exfoliation of expanded graphite with thicknesses in the range 1–10 nm, and lateral linear dimensions varying from about 1 μm up to 10 μm [20,21]. GNPs are 2D-carbon nanostructures typically characterized by a greater thickness (in the range 20–80 nm) and lateral dimensions in the micron range [13]. From the EM point of view, both MLGs and GNPs can play a dual role when dispersed inside a polymer matrix. In fact, the nano-metric size of these nanostructures mainly influences the electron transport properties of the composite, whereas the wide surface area in the micron-range mainly affects dielectric polarizability. Therefore, the control of both the thickness and the lateral dimensions of the nanofiller is a crucial point in the production of high performance RAMs. 

A general review of the fabrication, properties, and uses of graphene-based composites is reported in [17], focusing on different polymer matrices (such as epoxy, polyvinylidene fluoride , poly methyl methacrylate , polyurethane , polyvinyl chloride , etc.) and considering either GOs, reduced-GOs (RGOs), or GNPs as fillers. An extensive review of the microwave properties of polymer composites filled with different carbonaceous nanomaterials with different shapes is reported by Qin and Brosseau in [15], with particular emphasis on the use of such composites as radar absorbing materials (RAMs). In their work, they highlight the numerous factors that affect the electrical and microwave properties of carbon-based polymer composites, one of them being the degree of aggregation or agglomeration of the nanofiller.

The formation of nanofiller aggregates in a composite can strongly degrade the functional properties of the final material (from both the electrical and mechanical points of view) for several reasons. Firstly, when aggregates are present in a composite, the nanometer scale of the filler material is lost and, consequently, the effective electrical conductivity of the composite increases. Moreover, the percolation threshold of the composite increases, since the aspect ratio of the inclusions (i.e., nanofiller aggregates) tends to unity. In addition, macro-sized aggregates can behave as mechanical defects, originating cracks in the composite when subjected to a mechanical strain that overcomes the van der Waals forces binding nanostructures within the aggregate together. For these reasons, the control of nanofiller distribution and aggregate formation inside the composite when using in particular MLGs or GNPs as filler material is a challenge, and should be avoided during the manufacturing process [22,23,24].

In general, in a solution-based process, the nanofiller is firstly dispersed in a proper solvent and successively the colloidal suspension is mixed with the polymeric matrix; in this case, the Hansen parameters of the suspension can be adjusted in order to match those of the polymer in liquid phase in order to enhance nanofiller dispersion and integration [25]. The production of MLG-filled nanocomposite through the solution process has been widely investigated in the literature [15,16,17,18,26,27,28,29,30]. One process [26,27,28] consists of the liquid-phase exfoliation of thermally-expanded graphite so as to obtain MLGs, the subsequent mixing of the MLG-based suspension with the polymer, followed by solvent evaporation and final curing of the resulting solvent-free mixture. However, solvent evaporation during composite processing and curing often represents a real bottleneck in the solution-based approach, because it can cause polymer chain disruption and mechanical property degradation of the final material. For this reason, solvent-free strategies are often preferred to solution-based processes, even if good nanofiller dispersion and aggregate prevention represent an extra challenge. 

The assessment of the presence of nanofiller aggregation in a composite is typically performed through microscopy investigations (TEM, SEM, AFM), X-ray analysis (XRD), nuclear magnetic resonance (NMR), infra-red or Raman spectroscopy [29]. However, these techniques are quite inefficient in the case of large-scale applications, since in general they provide only local information about the aggregation state of the nanofiller rather than an effective representation of the effects of aggregate formation on the functional property degradation of the composite.

The present study is focused on the investigation of the electrical, electromagnetic, and dynamic mechanical properties of epoxy-based composites filled with GNPs. We propose an innovative approach to assess the formation of aggregates in the composite on a large-scale and to estimate their average size. The method is based on the measurement of the effective complex dielectric permittivity of the composite at radio-frequency (i.e., in the x-band ranging from 8 to 12 GHz) and on the application of the Multiscale Maxwell Garnet (MMG) model, which was developed and validated [26,27] for the effective medium modelling of GNP-based polymer composites. The MMG method allows one to account for the combined effects of the 2D-shape of the GNPs and the electromagnetic scattering produced by their sharp edges and surface wrinkles on the complex effective permittivity of the composite. In this work, the MMG approach is applied to evaluate the effective size of the aggregates from the best fit of the experimental data. The results of this analysis are consistent with the composite percolation behaviour, obtained both through experimental measurements and calculations based on prediction models available in the literature.

In this paper, we develop a solvent-free process for the production of graphene-based composites, using two different types of commercial thermosetting polymer resins as matrix, and GNPs as filler. The morphological properties of the nanofiller are initially investigated using atomic force microscopy (AFM) and field-emission scanning electron microscopy (FE-SEM). FE-SEM analyses are also performed in order to assess (at the micro-scale) dispersion and integration of the nanofiller in the polymer matrix, as well as aggregate formation with increasing nanofiller concentration. Therefore, full characterization of the dynamic mechanical, electrical, and electromagnetic properties of the produced nanocomposites was undertaken. We demonstrate that the cross-comparison of the results of FE-SEM investigations, dynamic mechanical thermal analysis (DMTA), and complex permittivity measurements in the x-band represent a powerful tool to assess the presence of filler agglomerations. In particular, from the numerical fitting of the EM test results through the use of the MMG model proposed in [26,27], we can obtain an estimate of the average size of the filler agglomerations in the produced nanocomposites. The proposed method is particularly suitable for large-scale application of these composites, such as in aeronautical stealth technology.

## 2. Materials and Methods

### 2.1. Materials

Epoxy (Epikote LR135) and epoxy-based vinyl (DION 9102) ester resins, produced by Momentive (Columbus, OH, USA) and Reichhold (Durham, NC, USA), respectively, were used as polymeric matrices. The epoxy resin system did not contain solvents or additives and had a density between 1.14 g/cm^3^ and 1.18 g/cm^3^, with a viscosity in the range of 2300–2900 mPa·s. The vinyl ester resin had a viscosity of 150–200 mPa·s and a density between 1.01–1.05 g/cm^3^, with a styrene content of around 50% wt.

**Figure 1:**
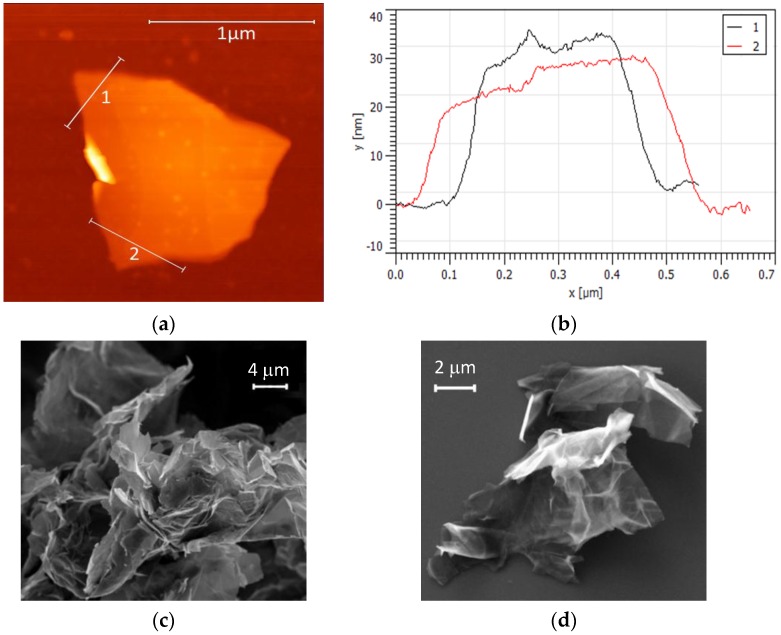
(**a**) AFM image of graphene nanoplatelets (GNPs) and (**b**) corresponding thickness profiles. (**c**,**d**) field emission-scanning electron microscope (FE-SEM) images of GNP powders at different magnifications.

GNPs were used as nanofiller. The morphology of the GNPs was analysed using a Bruker-Veeco Dimension Icon AFM (Bruker Corporation, Billerica, MA, USA) operated in tapping mode and a Zeiss Auriga FE-SEM (Carl Zeiss, Oberkochen, Germany), both available at Sapienza Nanotechnology and Nanoscience Laboratory (SNN-Lab) (Roma, Italy). Figure 1a,b show the height signal mapping over a GNP flake surface and two selected thickness profiles extracted from the same image. Figure 1c,d show FE-SEM images of GNP flakes at different magnifications.

From these observations, it can be concluded that the nanofiller consists of large aggregates of small nanoplatelets, with lateral sizes of up to a few microns and a thickness between 20–60 nm. The aggregates can reach lateral dimensions of up to a few tens of microns.

**Figure 2:**
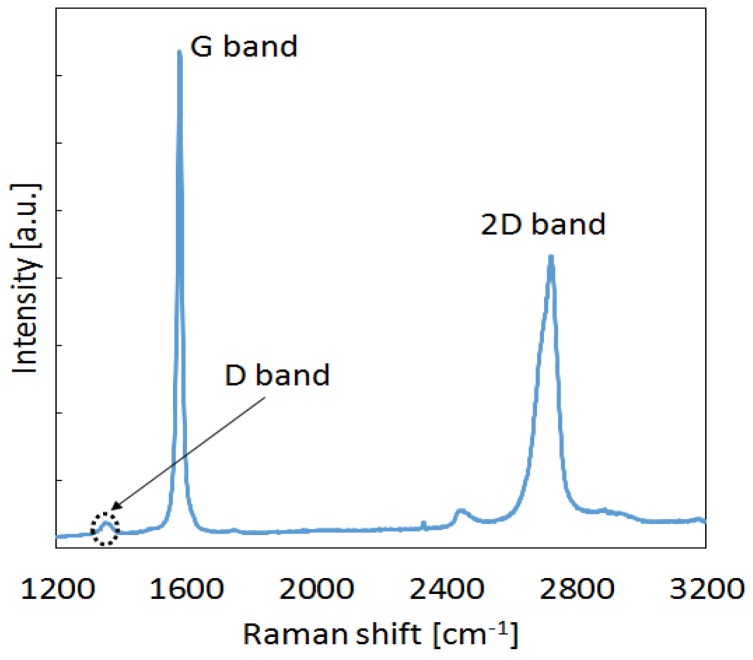
Raman spectrum of the commercial GNPs.

A Renishaw In-Via Reflex Raman System (Renishaw plc, Wotton-under-Edge, United Kingdom) coupled to an optical microscope was used to evaluate the structural properties of the commercial nanofiller. The Raman scattering was excited using an Argon ion laser with a wavelength of 514.5 nm, focused on the sample with a 50× microscope objective, with a laser power at the sample of ~50 mW, employing an exposure time of 10 s and three accumulations over the spectral range 1200–3200 cm^−1^. Figure 2 shows a representative Raman spectrum of the commercial GNP powder. It presents two strong bands: the sharp G band at around 1570 cm^−1^, which is characteristic of the sp^2^ type bonding of the carbon atoms in the basal plane, and a second order 2D band at 2700 cm^−1^, which is sensitive to the number of stacked graphene layers in the platelet. This number can be estimated from the shape of the curve and the relative intensity of its peak [31]. 

In this case, both the observed bandshape and the intensity ratio (*I*_2D_/*I*_G_) of ~0.63 indicate that there are relatively few layers in the stack. Another characteristic band in the Raman spectrum of graphene and graphitic materials is the D-band, observed here as a very small peak at ~1350 cm^−1^, and is associated with disordered sp^3^-hybridized carbon impurities or defects in the pristine graphene structure of GNPs [31]. The very low intensity ratio (*I*_D_/*I*_G_) of 0.05, and the virtual absence of the D’ band—ascribed to edge defects and habitually encountered at around 1620 cm^−1^—is representative of a well-ordered structure.

### 2.2. Production Process

GNP-filled composites were fabricated at SNN Lab (Roma, Italy), following two different solvent-free procedures that have the main advantage (with respect to liquid-phase mixing) of reducing the possibility of polymer structural damage. The samples were produced at different GNP filler concentrations from 0.5% to 3% wt.

**Figure 3:**
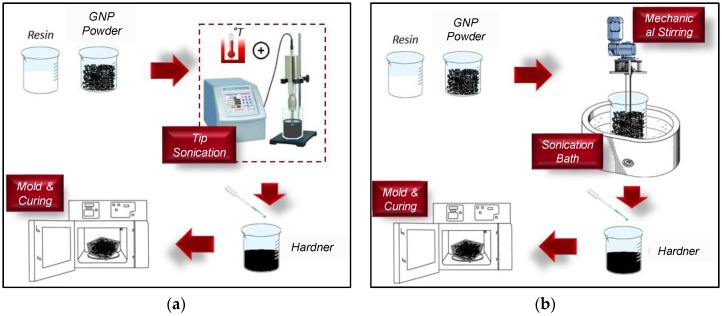
Sketch of GNP-composite production processes: (**a**) epoxy-based system; and (**b**) vinyl ester-based system.

GNP composites based on epoxy resin were produced following the process illustrated in Figure 3a, consisting of the use of a jacketed beaker and an ultra-sonicating tip. The nanofiller and resin were added into the jacketed beaker at 70 °C and homogenized using a Sonics VC 505 tip sonicator (The Sonics, Tacoma, WA, USA) in pulse mode (1 s on/1 s off) and an amplitude of 40% for a total time of 40 min. Subsequently, the hardener was added to the mixture, which was then stirred for a further 10 min. The final mixture was poured into aluminum rectangular flanges and cured for 24 h at room temperature, followed by a post-cure regime of 24 h at 70 °C.

The epoxy-based vinyl ester resin was filled with commercial GNP powders following the steps in Figure 3b, which include a combination of bath sonication and mechanical stirring instead of the aforementioned tip sonication. This choice was mainly suggested by the need to limit resin overheating and styrene evaporation during processing. At first, the resin mixed with the GNP powders was bath sonicated for 4 h at 25 °C, and was then mechanically stirred for 30 min in order to obtain a homogeneous suspension. After the addition of the hardener, the mixture was finally poured into the flanges, following the same procedure used for the epoxy-based composites.

### 2.3. GNP-Composite Characterization

In order to investigate the uniformity of the nanofiller dispersion in the polymer matrix, an extensive FE-SEM study was undertaken, using a Zeiss Auriga FE-SEM available at SNN-Lab. Images were obtained from cryogenically fractured samples bonded to SEM stubs using carbon double sided adhesive tape and sputtered with a chromium coating.

Dynamic Mechanical Thermal Analysis (DMTA) measurements were undertaken using a Mettler DMA861e (Mettler-Toledo, Greifensee, Switzerland). Each sample was tested at different frequencies (1 Hz, 5 Hz, 10 Hz, and 30 Hz) in order to investigate the behaviour of the materials at different stress intensities, and the results are presented in terms of the storage modulus, *M*’, and of the loss factor, tanδ, as a function of the temperature, *T*.

The DC electrical conductivity and the complex dielectric permittivity of the nanocomposites cured inside rectangular flanges were measured at room temperature in the Electromagnetic Compatibility Lab of the Department of Astronautics, Electrical and Energy Engineering at Sapienza University (Roma, Italy). The effect of temperature on the electromagnetic properties of the composites was discussed elsewhere [30], and is not within the scope of this paper.

The effective DC electrical conductivity $\sigma_{DC}$ was extracted from the resistance values measured by applying the two-wire volt-amperometric method. To this purpose, the opposite faces of each sample were coated with silver conductive paint (Electrolube, Ashby de la Zouch, United Kingdom) and dried at 70 °C for 10 min. Then, tin-coated copper wires were bonded to the aforementioned faces using a bi-component Ag-filled epoxy adhesive (Circuitworks, Kennesaw, GA, USA). Subsequently, the samples were oven cured at 120 °C for 10 min. The samples filled with GNP at 1.5%, 2%, 2.5%, and 3% wt. were electrically characterized using a Keithley 6221 DC/AC current source (Keithley Instruments, Solon, OH, USA) connected to a Keithley 2182a nano-voltmeter, controlled by a laptop. The samples with GNP concentration below 1.5% wt, due to high electrical resistivity, were tested using a Keithley 6517B electrometer (Keithley Instruments, Solon, OH, USA). Finally, the effective DC electrical conductivity $\sigma_{DC}$ was extracted from the measured resistance values.

The scattering parameters of brick-shaped samples were measured with a vector network analyser (Anritsu Vector Star MS4647A) (Anritsu, Kanagawa, Japan) in the X- band, covering the frequency range 8.2–12.4 GHz. The rectangular aluminum flange has dimensions of 22.86 mm × 10.16 mm × 6 mm. The surface of the samples was accurately finished using a polishing machine (Buehler, Lake Bluff, IL, USA). All samples were dried for 24 h at controlled temperature and humidity. For each composite type, a batch consisting of three flanges was prepared. A total of three different measurements were performed for each nanocomposite formulation, and data was averaged in order to reduce uncertainty. The complex effective permittivity of the nanocomposites $\left({\varepsilon^{\prime }}_{r}+j{\varepsilon^{\prime \prime }}_{r}\right)$ was finally extracted from the measured parameters following a standard method [32].

## 3. Results

### 3.1. SEM Characterization of Composite Samples

**Figure 4:**
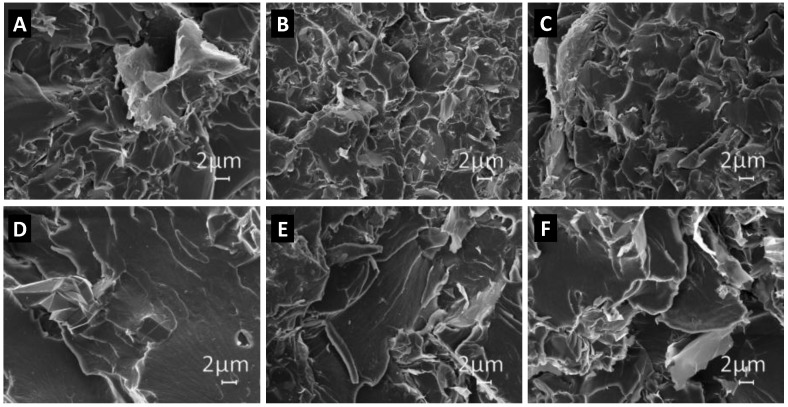
SEM images of the fractured surface of epoxy (**A**–**C**) and vinyl-ester (**D**–**F**) carbon-based nanocomposites, filled with GNPs at different concentrations: (**A**,**D**) 0.5 wt %, (**B**,**E**) 1 wt %, (**C**, **F**) 2 wt %.

Figure 4 shows SEM images (with a magnification of 5000×) of the composites filled with different weight concentrations of GNPs and produced using the two different polymer matrices: the epoxy system (top row) and the vinyl-ester one (bottom row). We notice that the filler distribution within the composite is quite uniform in all cases, even the formation of aggregates with an average size from a few up to >10 µm is evident. It is also observed that in the epoxy-composites, the average size of the filler aggregate is smaller than in the vinyl-ester case. This is probably due to the effect of the tip-sonication step (as shown in Figure 3a) used for mixing GNPs and resin in the case of epoxy-based composites.

### 3.2. DMTA

DMTA measurements were performed in order to assess the mechanical properties of the produced nanocomposites.

**Figure 5:**
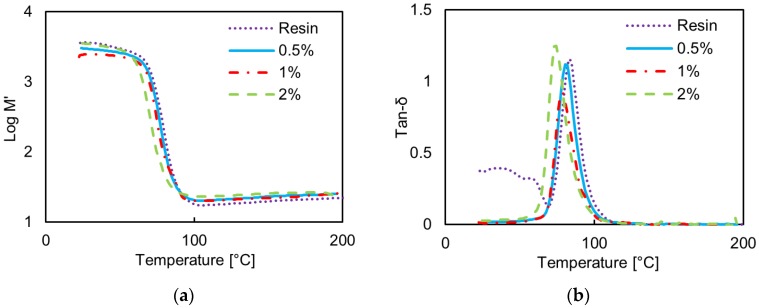

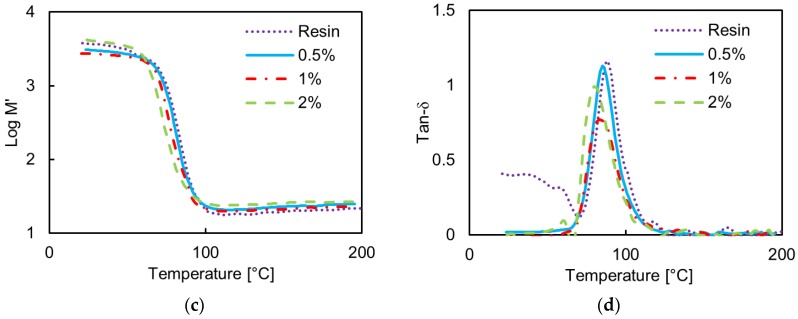
Measured storage modulus *M*’ and loss tangent tanδ vs. temperature of the neat epoxy resin and of the epoxy-based composites filled with GNPs at increasing wt. % concentration: (**a**) *M*’ and (**b**) tanδ at 5 Hz; (**c**) *M*’ and (**d**) tanδ at 30 Hz.

Figure 5 shows the measured storage modulus, *M*’, and the loss tangent, tanδ, for the epoxy-based GNP composites at 5 Hz and 30 Hz. A little improvement in the storage modulus is noticed below the transition temperature *T*_g_ corresponding to the peak of tan δ, whereas above *T*_g_ only a slight improvement is found. On the other hand, *T*_g_ falls monotonically with increasing GNP content, from 83 °C at 5 Hz in the case of the neat resin to 74 °C at 5 Hz in the case of the composites filled with 2 wt %. GNP. An increase in *T*_g_ would be indicative of good adhesion between the epoxy matrix and the nanoplatelets, resulting in an increased rigidity as the segmental motion of the crosslinks in the matrix under loading is restricted by the nanofillers. However, in this case, the decrease in *T*_g_ is indicative of the tendency of the nanofiller to agglomerate with increasing concentration. Further, we notice that the width of the tanδ peak is slightly increased at the highest GNP concentration of 2 wt %: this is also indicative of a more heterogeneous filler distribution [33].

**Figure 6:**
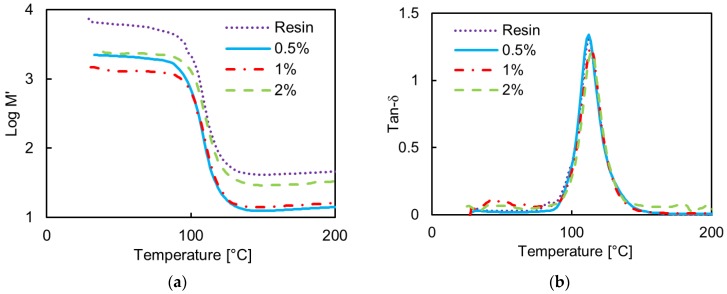

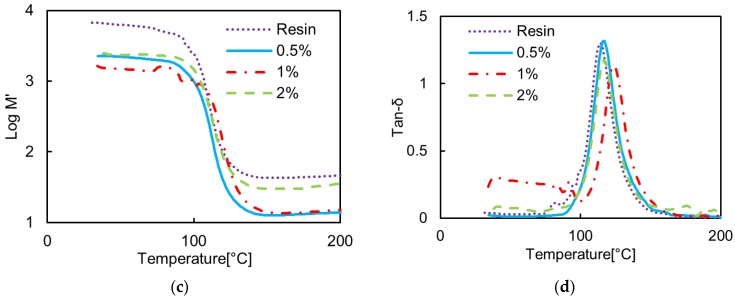
Measured storage modulus *M*’ and loss tangent tanδ vs. temperature of the neat vinyl-ester resin and of the vinyl-ester-based composites filled with GNPs at increasing wt % concentration: (**a**) *M*’ and (**b**) tanδ at 5 Hz; (**c**) *M*’ and (**d**) tanδ at 30 Hz.

Figure 6 shows the measured storage modulus, *M*’, and the loss tangent, tanδ, at the frequencies of 5 Hz and 30 Hz of the composites produced using the vinyl ester resin. We notice a significant decrease of the storage modulus with respect to the neat resin, regardless of the test frequency. The chemical composition of the resin has a very high styrene content (approximately 50 wt %.), and the mechanical properties of the corresponding cross-linked composites are largely affected by the residual styrene content, which in turn is affected by the manufacturing process of the composite [34]. In this case, the sonication step during the production process (Figure 3b) involves a loss of styrene (vapour pressure of 0.67 KPa at 20 °C), which leads to a reduction in the density of cross-linking sites, resulting in a lower modulus. Thus, we can assume that two main counteracting mechanisms are involved in the observed modification of mechanical properties of the vinyl ester composite, as discussed in the following.

The first factor is related to the high Young’s modulus of the nanofiller, which should contribute to an increase in the storage modulus of the composite and to a corresponding increase in the glass transition temperature (*T*_g_). The second factor is related to the loss of styrene during filler mixing and material processing, which contributes to a reduction in the Young’s modulus of the final composite. The latter effect is the most significant in this system. For lower filler concentrations (0.5 wt % and 1 wt %), we observe a reduction of *M*’ because the styrene loss during processing is the dominant mechanism. Simultaneously, we observe an increase in *T*_g_ with filler concentration up to 1 wt %. The increase is more evident at higher frequency (30 Hz): this is a confirmation of a better adhesion and more uniform GNP dispersion in the vinyl-ester composites than in the epoxy-based ones. On the contrary, for the highest filler concentration of 2 wt %, the relative increase in *M*’ is justified by the high GNP concentration, which balances out the effect of styrene loss during material production. In this case a reduction in the *T*_g_ is observed, clearly associated with the presence of GNP aggregates.

### 3.3. DC Conductivity Measurement

**Figure 7:**
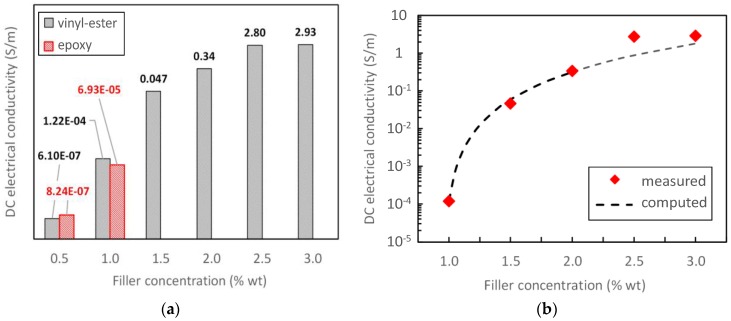
(**a**) Measured DC electrical conductivity of epoxy and vinyl-ester-based composites filled with GNPs at increasing wt. % concentration; (**b**) Estimated percolation curve of the vinyl-ester composites filled with GNPs: $\theta_{c}=0.95\%,\;K=42.5\frac{kS}{m},\;t=2.58.$

Figure 7a shows the measured DC electrical conductivity $\sigma_{DC}$ of the GNP-composites, produced using either the epoxy or the vinyl-ester resin, as a function of GNP content. With regard to the epoxy-based composites, the measured DC conductivity at filler concentrations higher than 1% wt. are not displayed because of the evident presence of agglomerates, already noted from the DMTA results, which do not make them consistent with those obtained at lower concentrations. With regard to the vinyl-ester composites, we notice an increasing value of the DC electrical conductivity for increasing filler concentration, up to a value of ~3 S/m at 3 wt %.

From the analysis of the measured vinyl-ester composite DC conductivity data, we can assume that the percolation threshold of the composite is around the GNP concentration of 1 wt %. Therefore, we obtain the percolation curve as the best fit of the measured DC conductivities of the composite specimens with a GNP concentration near the estimated percolation threshold (i.e., from 1 wt % to 2 wt %) using the power law formula in [35,36]:

(1) $$\sigma_{DC}=K{\left(\theta -\theta_{c}\right)}^{t}$$

in which θ is the weight concentration of the filler in percent, $\theta_{c}=0.95\%$ is the estimated percolation threshold expressed as filler weight concentration in percent, t=2.59 is the critical exponent, and K=42.5 kS/m is a dimensional constant. The mean error of the fit in the range 1%–2 wt % is 0.48 dB, with a standard deviation of 0.37 dB. For higher concentrations, the fitting error is higher because the power law (1) is valid for $0<\left(\theta -\theta_{c}\right)<1$ [36].

### 3.4. Complex Permittivity Measurement

**Figure 8:**
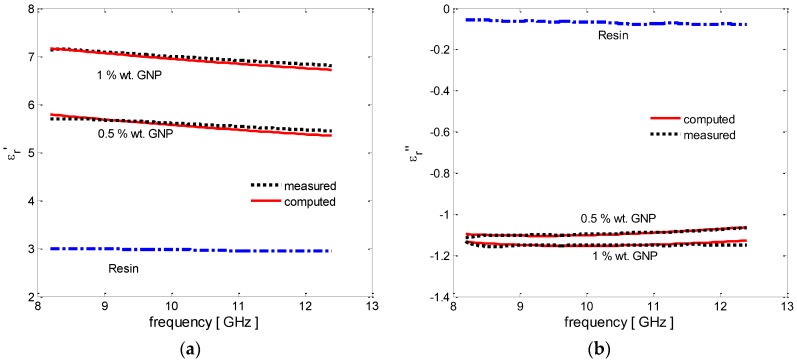
Complex effective permittivity of the GNP-filled epoxy-based composites with increasing filler concentrations: (**a**) real part and (**b**) imaginary part.

**Figure 9:**
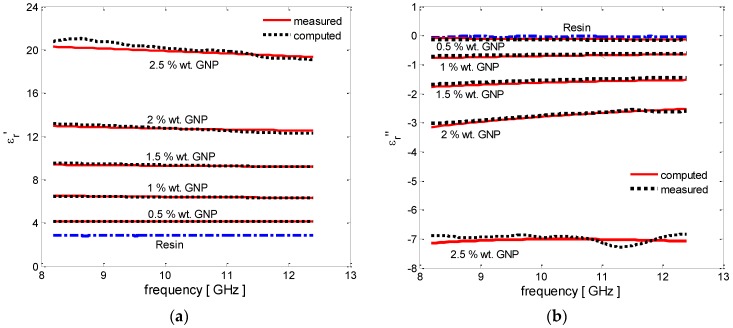
Complex effective permittivity of the GNP-filled vinyl ester-based composites with increasing filler concentrations: (**a**) real part and (**b**) imaginary part.

The measured spectra of the real and imaginary parts of the complex effective permittivity of the epoxy resin and vinyl-ester resin-based GNP-filled composites are shown in Figure 8 and Figure 9 (dotted line), respectively.

We notice that the GNP/epoxy nanocomposites are characterized by an increase in the values of the modulus of the real and imaginary parts of the complex effective permittivity (${\varepsilon^{\prime }}_{r}\;\text{and}\;{\varepsilon^{\prime \prime }}_{r},\;\text{respectively}$) for filler concentrations up to 1 wt %. The measured relative permittivity of the composites filled at higher weight concentration are not displayed because of the evident presence of agglomerates, already noted from the DMTA results, which does not make them consistent with the ones obtained at lower concentration. This suggests that the dispersion method should be improved.

On the other hand, for the full series of vinyl ester composites, we observe an increase in the modulus of the real and imaginary parts of the complex effective permittivity as a function of the filler concentration, as seen in Figure 9a,b.

## 4. Discussion

SEM, DMTA, DC conductivity, and complex dielectric permittivity measurements all indicate the formation of GNP aggregates within the composites, with different degrees of agglomeration that vary with filler concentration. The average size of these aggregates increases with filler concentration, and depends on the type of polymer matrix and on the composite manufacturing process. In the following section, we show how it is possible to estimate the average size of nanofiller aggregates through the analysis of the data resulting from the complex effective permittivity measurement of the composite. Moreover, we use the results of this analysis to discuss which are the main factors contributing to electrical conductivity in the composite.

### 4.1. Aggregate Average Size Estimation through MMG Modelling

The effective medium approach has been widely investigated in the literature by several authors [37,38,39,40,41,42,43] in order to predict the electromagnetic properties of polymer composites. In fact, whereas ab-initio [42] and finite element [43] computational techniques can be used to model the filler–matrix interaction at the nano- and micro-scale and its effect on the electrical and electromagnetic properties of the composite, the effective medium theory allows modelling of the composite as a homogeneous effective medium, characterized by the same complex dielectric permittivity at radio-frequency of the composite [37,38]. In particular, the Maxwell Garnet (MG) model has been largely applied in order to predict the complex effective permittivity of epoxy or vinyl-ester composites filled with short carbon fibers, CNTs, and multiphase systems [39,40,41]. However, it should be pointed out that the models available in the literature generally assume that the filler has an ellipsoidal shape. This means that, with regard to GNP-filled composites, the effects of EM field scattering, localizations, and enhancements due to the irregular shape of the graphene flakes cannot be properly taken into consideration. In order to overcome such limitations, the MMG model was proposed [26,27] in order to properly take into account the 2D-irregular shape of GNPs, characterized by sharp edges and wrinkles that contribute to the polarization and conducting properties of the composites at radio-frequency and microwave. Thus, in the MMG approach, a GNP-filled composite is modelled as a hierarchic bi-filler composite. Initially, a first effective medium, containing an effective filler made of oblate ellipsoids dispersed in the plain polymer matrix is defined. Then, a second effective medium, containing a second effective filler made of nanorods dispersed in the first effective medium, is considered. In fact, it was observed that graphene flakes can be circumscribed by oblate ellipsoids [26], and that electric field localizations and enhancements produced by wrinkles characterizing the GNP surface and by the sharpness of the GNP edges can be considered as the effect of scattering and polarization generated by nanorods [26]. In the considered frequency range up to 10–20 GHz, both effective fillers are assumed to be conductive with a negligible dielectric permittivity. In fact, recent studies have shown that the frequency relaxation time of GNPs is about 5 THz [44]. Moreover, in-depth studies on graphene electrical properties have shown that in the considered frequency range, the real part of graphene conductivity is almost constant and the imaginary part can be neglected [45].

In the following, we summarize the MMG approach, considering that the composite consists of a polymer matrix filled with micro-sized inclusions made of GNP agglomerates. The average size of these agglomerates (average lateral dimension and average thickness) is estimated through the best fit of the MMG model to the measured complex permittivity data. 

At first, we compute the effective permittivity of the polymeric matrix filled with oblate ellipsoids having axes $l_{\text{OBL}}$, $t_{\text{OBL}}$, and electrical conductivity $\sigma_{\text{OBL}}$, where $l_{\text{OBL}}$ is the average lateral dimension of the GNP agglomerates and $t_{\text{OBL}}$ their average thickness, resulting in the following equation:

(2) $$\varepsilon_{\text{rOBL}}=\varepsilon_{\text{rP}}+\frac{\varepsilon_{rP}\theta_{OBL}\left[1-\varepsilon_{rP}-j\sigma_{OBL}/\left(\omega \varepsilon_{0}\right)\right]\;\sum_{k=1}^{3}\Lambda_{kOBL}}{3-\theta_{OBL}\left[1-\varepsilon_{rP}-j\sigma_{OBL}/\left(\omega \varepsilon_{0}\right)\right]\;\sum_{k=1}^{3}\Gamma_{kOBL}}$$

with:

(3a) $$\Lambda_{\text{kOBL}}=\frac{1}{\varepsilon_{\text{rP}}+N_{\text{kOBL}}\left[1-\varepsilon_{\text{rP}}-j\sigma_{\text{OBL}}/\left(\omega \varepsilon_{0}\right)\right]}\;k=1,3$$

(3b) $$\Gamma_{\text{kOBL}}=\frac{N_{\text{kOBL}}}{\varepsilon_{\text{rP}}+N_{\text{kOBL}}\left[1-\varepsilon_{\text{rP}}-j\sigma_{\text{OBL}}/\left(\omega \varepsilon_{0}\right)\right]}\;k=1,3$$

where $\varepsilon_{\text{rP}}$ is the relative dielectric constant of the polymer matrix and $\theta_{\text{OBL}}$ is the volume fraction of the oblate ellipsoid, which is assumed equal to the GNP volume fraction. $\theta_{\text{GNP}}$ and $N_{\text{kOBL}}$ (*k* = 1, 3) are the depolarization factors of oblate ellipsoids randomly dispersed in a uniform medium:

(4a) $$N_{1\text{OBL}}=N_{2\text{OBL}}=\frac{\left(1-N_{3\text{OBL}}\right)}{2}$$

(4b) $$N_{3\text{OBL}}=\frac{\left(1+e_{\text{OBL}}^{2}\right)\left[e_{\text{OBL}}-tan^{-1}\left(e_{\text{OBL}}\right)\right]}{e_{\text{OBL}}^{3}}$$

$e_{OBL}$ being the eccentricity of the oblate ellipsoid having maximum and minimum diameters of $l_{OBL}$ and $t_{OBL}$:

(5) $$e_{\text{OBL}}=\sqrt{{\left(\frac{l_{\text{OBL}}}{t_{\text{OBL}}}\right)}^{2}-1}$$

Successively, the MG expression is iteratively applied, considering that rod-shaped inclusions are dispersed in the effective medium constituted by the matrix loaded with the oblate ellipsoids having an effective permittivity $\varepsilon_{\text{rOBL}}$ given in (2). This yields:

(6) $$\varepsilon_{\text{rEFF}}=\varepsilon_{\text{rOBL}}+\frac{\varepsilon_{rOBL}\theta_{ROD}\left[1-\varepsilon_{rOBL}-j\sigma_{ROD}/\left(\omega \varepsilon_{0}\right)\right]\;\sum_{k=1}^{3}\Lambda_{kROD}}{3-\theta_{ROD}\left[1-\varepsilon_{rOBL}-j\sigma_{ROD}/\left(\omega \varepsilon_{0}\right)\right]\;\sum_{k=1}^{3}\Gamma_{kROD}}$$

with:

(7a) $$\Lambda_{\text{kROD}}=\frac{1}{\varepsilon_{\text{rOBL}}+N_{\text{kROD}}\left[1-\varepsilon_{\text{rOBL}}-j\sigma_{\text{ROD}}/\left(\omega \varepsilon_{0}\right)\right]}\;k=1,3$$

(7b) $$\Gamma_{\text{kROD}}=\frac{N_{\text{kROD}}}{\varepsilon_{\text{rOBL}}+N_{\text{kROD}}\left[1-\varepsilon_{\text{rOBL}}-j\sigma_{\text{ROD}}/\left(\omega \varepsilon_{0}\right)\right]}\;k=1,3$$

In the previous expressions, it results that $\theta_{\text{ROD}}=\alpha_{\text{ROD}}\theta_{\text{GNP}}$ and $\sigma_{\text{ROD}}=\beta_{\text{ROD}}\sigma_{\text{GNP}}$, in which $\alpha_{\text{ROD}}$ and $\beta_{\text{ROD}}$ are representative of the relative concentration of scattering edges characterizing the GNP agglomerates and of their relative effective electric conductance with respect to GNPs. The depolarization factors $N_{\text{kROD}}$ (*k* = 1, 3) are those of cylindrical rods having length $l_{ROD}$ and diameter $t_{\text{ROD}}$, randomly dispersed in a uniform medium:

(8a) $$N_{1\text{ROD}}=N_{2\text{ROD}}=0.5$$

(8b) $$N_{3\text{ROD}}={\left(\frac{t_{\text{ROD}}}{l_{\text{ROD}}}\right)}^{2}ln\left(\frac{l_{\text{ROD}}}{t_{\text{ROD}}}\right)$$

in which $l_{\text{ROD}}=l_{\text{OBL}}$, $t_{\text{ROD}}=\gamma_{\text{ROD}}t_{\text{OBL}}$, and $\gamma_{\text{ROD}}$ is a parameter that takes into account the effects of the production process on the resulting morphology of GNPs.

The numerical fitting of the EM test results through the use of the MMG model can thus provide an estimate of the average size of the filler agglomerations in the produced nanocomposites, which is in line with the SEM analysis and DTMA observations. The results obtained—shown in Figure 8 and Figure 9 and reported in Table 1 and Table 2—demonstrate that the measured complex effective permittivity are well approximated by the MMG model, assuming a constant value of the electrical characteristics of the two effective fillers (the oblate ellipsoids and the rods) inside the effective medium (i.e., $\sigma_{\text{OBL}}\;\text{and}\;\sigma_{\text{ROD}}$) for increasing weight concentration of GNPs and for the two different resin systems (i.e., epoxy and vinyl ester). On the contrary, the average dimensions of the two effective fillers (i.e., $l_{\text{OBL}},\;t_{\text{OBL}},\;l_{\text{ROD}},\;t_{\text{ROD}}$) increase as the GNP weight concentration increases, due to the different degrees of aggregation of composites with different filler concentrations, and differ for the two resin systems due to the variations in the manufacturing process.

In particular, GNP aggregates in the epoxy system are characterized by smaller lateral dimensions and average thickness with respect to those in the vinyl ester. This can be ascribed to the use of tip sonication in the epoxy-composite processing instead of mechanical mixing in the vinyl ester case. In fact, tip sonication produces a higher exfoliation of graphite, but at the same time a reduction of the lateral size of the flakes due to the effect of the disruptive energy released in the fluid during cavitation. The aspect ratio of the aggregates (i.e., $l_{\text{OBL}}/t_{\text{OBL}}$) is in the range 118–133 for GNP concentrations between 0.5 wt % and 1 wt %.

The vinyl ester composite is characterized by GNP aggregates with average dimension of up to ~30 μm for GNP concentration of 2.5 wt %. The aggregate aspect ratio ranges from a minimum of ~118 to a maximum of ~430. The higher value of the aggregate aspect ratio in the vinyl ester composites is in line with the results of the EM characterizations, which show higher modulus of the real and imaginary parts of the complex effective permittivity in the vinyl-ester composites than in the epoxy-based ones. The same result is also confirmed by SEM analysis (Figure 4) and implied from DTMA (Figure 5 and Figure 6).

### 4.2. Effect of Filler Aggregation on the DC Electrical Conductivity of the Composite

In order to estimate the effect of filler aggregation on the DC electrical properties of the composite, we calculated the percolation thresholds of composites filled either with oblate ellipsoids or with cylindrical rods, having the geometrical characteristics reported in Table 1 and Table 2.

In general, the geometrical percolation threshold (expressed in volume fraction of filler) of a polymer composite filled with either oblate ellipsoids $\left(f_{\text{cOBL}}\right)$ or cylindrical rods $\left(f_{\text{cROD}}\right)$ is given by:

(9a) $$f_{\text{cOBL}}=k_{\text{OBL}}\frac{t_{\text{OBL}}}{l_{\text{OBL}}}$$

(9b) $$f_{\text{cROD}}=k_{\text{ROD}}\;\frac{t_{\text{ROD}}}{l_{\text{ROD}}}$$

in which the constants $k_{OBL}$ and $k_{ROD}$ take into account the effects of filler distribution, composite processing, and polymer interaction with the filler at the interface. Garboczi et al. [46] assumed $k_{\text{OBL}}=1.27$ and $k_{\text{ROD}}=0.6$, mainly on the basis of pure geometrical considerations. Neelakanta [38] and Lagarkov et al. [39] come to the conclusion that $k_{ROD}$ has a value between 1 and 4.5, depending on the arrangement of the rods in the composite. The barrier effect at the interface between oblate ellipsoids and the polymer matrix is taken into account by Lu and Mai [47], who propose $k_{\text{OBL}}$ = 2.154. The corresponding values of percolation threshold expressed in wt % are given by:

(10) $$\theta_{\text{cOBL}\left(\text{ROD}\right)}=100\times \frac{\delta_{\text{GNP}}}{\delta_{\text{res}}}\left(\frac{f_{\text{cOBL}\left(\text{ROD}\right)}}{1-f_{\text{cOBL}\left(\text{ROD}\right)}}\right)$$

in which $\delta_{\text{GNP}}\;$= 2.15 kg/cm^3^ and $\delta_{\text{res}}\;$= 1.20 kg/cm^3^ are the values of the mass densities of GNPs and resin used in this study, respectively.

Table 3 and Table 4 show the values of percolation thresholds (in wt %) computed for composites filled with either oblate ellipsoids or cylindrical rods having the geometrical characteristics reported in Table 1 and Table 2 and resulting from the best fit of the measured complex permittivity data obtained using the MMG method. 

It is noted that for both composite types (epoxy- and vinyl ester-based), the percolation effect is dominated by the oblate-like shape of the GNP aggregates: in fact, the values of the calculated percolation threshold of the oblate-ellipsoid-filled composite (see column $\theta_{\text{cOBL}}$ in Table 3 and Table 4) are comparable to or lower than the effective concentration of the oblate ellipsoids in the composite (see data in column $\theta_{\text{OBL}}$). On the contrary, the values of the calculated percolation threshold of the rod-filled composite (see column $\theta_{\text{cROD}}$ in Table 3 and Table 4) are much higher than the effective concentration of the rods in the composite (see data in column $\theta_{\text{ROD}}$). Moreover, results show that the epoxy-based composite is probably characterized by a percolation threshold slightly higher than 1 wt %, whereas the vinyl-ester-based composite is percolating above 1 wt %, as was also observed in Section 3.3 (Figure 7b). This conclusion coincides with the lower aspect ratio of the filler in the epoxy-based composites with respect to those of the vinyl-ester (see data in Table 1 and Table 2).

## 5. Conclusions

This paper describes the electrical, EM, and dynamic mechanical properties of thermosetting resins filled with commercial GNPs. The production processes are developed via a solvent-free procedure and are suitable for large-scale industrial exploitation. The main novelty of the proposed approach is the tentative implementation of solvent-free production processes, but at the same time estimating the impact that the formation of nanofiller aggregates has on the functional properties of the composite and the average size of these aggregates.

The electrical and EM characterizations show different dielectric and conductive properties in the composites produced with the two different resins. In particular, for the epoxy system it can be observed that at a lower filler concentration (up to 1 wt %) there is a slight increase in the modulus of the real and imaginary parts of the complex effective permittivity. For the vinyl ester-based composites, the increase of the modulus of both the real and imaginary parts of the complex permittivity is much more evident, due to the formation of larger GNP aggregates, as confirmed through SEM observations and EM modelling.

The results of the DMTA analysis are in line with the results of the EM characterizations. In particular composites produced from the epoxy resin show no evidence of improvement in modulus below the transition temperature *T*_g_. Moreover, the decrease in *T*_g_ is indicative of the tendency of the nanofiller to agglomerate for increasing concentration. In addition, the width of the peak of the loss tangent tanδ at the highest concentration is slightly increased; this is indicative of a more heterogeneous filler distribution. These results suggest a need to further optimize the process for filler concentrations higher than 1 wt %. On the other hand, the DMTA analysis of the vinyl-ester based materials show that the resulting properties of the composites can be mainly ascribed to two competing mechanisms: the styrene loss during processing and the presence of the filler. It is observed that the first mechanism is dominant for low filler concentration (up to 1 wt %), whereas for higher filler concentration, the second factor produced an improvement of the storage modulus of the nanocomposite.

## Figures and Tables

**Table 1:** Estimated fitting parameters of the Multiscale Maxwell Garnet (MMG) model applied to the measured complex effective permittivity of the epoxy-based composites produced in this study, as a function of the weight concentration of GNPs $\theta_{\text{GNP}}$.

$\theta_{\mathrm{GNP}}=\theta_{\mathrm{OBL}}$ (wt %)	$\sigma_{\mathrm{OBL}}$ (kS/m)	$l_{\mathrm{OBL}}$ = $l_{\mathrm{ROD}}$ (μm)	$t_{\mathrm{OBL}}$ (nm)	$\theta_{\mathrm{ROD}}$ (wt %)	$\sigma_{\mathrm{ROD}}$ (kS/m)	$t_{\mathrm{ROD}}$ (nm)
0.5	11	6.5	55	0.0925	5	88
1	11	8	60	0.045	5	90

**Table 2:** Estimated fitting parameters of the MMG model applied to the measured complex effective permittivity of the vinyl ester-based composites produced in this study, as a function of the weight concentration of GNPs $\theta_{\text{GNP}}$.

$\theta_{GNP}=\theta_{OBL}$ (wt %)	$\sigma_{OBL}$ (kS/m)	$l_{OBL}=l_{ROD}$ (μm)	$t_{OBL}$ (nm)	$\theta_{ROD}$ (wt %)	$\sigma_{ROD}$ (kS/m)	$t_{ROD}$ (nm)
0.5	11	7.2	61	0.065	5	134
1	11	15	70	0.045	5	140
1.5	11	28	65	0.105	5	143
2	11	23	70	0.14	5	154
2.5	11	29	73	0.193	5	164

**Table 3:** Percolation thresholds of composites filled either with oblate ellipsoids at different concentrations in wt % ($\theta_{\text{OBL}}$) or with cylindrical rods at different concentrations in wt % ($\theta_{\text{ROD}}$), having the geometrical dimensions reported in Table 1.

$\theta_{OBL}$ (wt %)	$\theta_{cOBL}$ (wt %)		$\theta_{ROD}$ (wt %)	$\theta_{cROD}$ (wt %)	
	Garboczi [46]	Lu [47]		Garboczi [46]	Lagarkov [39]
0.5	1.95	3.33	0.093	1.47	2.46
1	1.72	2.94	0.045	1.22	2.04

**Table 4:** Percolation thresholds of composites filled either with oblate ellipsoids at different concentrations in wt % ($\theta_{\text{OBL}}$) or with cylindrical rods at different concentrations in wt % ($\theta_{\text{ROD}}$), having the geometrical dimensions reported in Table 1.

$\theta_{OBL}$ (wt %)	$\theta_{cOBL}$ (wt %)		$\theta_{ROD}$ (wt %)	$\theta_{cROD}$ (wt %)	
	Garboczi [46]	Lu [47]		Garboczi [46]	Lagarkov [39]
0.5	1.95	3.33	0.065	2.026	3.4
1	1.07	1.82	0.045	1.009	1.69
1.5	0.53	0.9	0.11	0.55	0.92
2	0.7	1.18	0.14	0.72	1.21
2.5	0.57	0.98	0.19	0.61	1.021

## Abbreviations

AFM: Atomic Force Microscopy
CNT: Carbon nanotube
DMTA: Dynamic Mechanical Thermal Analysis
EM: Electromagnetic
FE-SEM: Field Emission Scanning Electron Microscopy
GHz: Giga-hertz
GNP: Graphene Nanoplatelet
GO: Graphene oxide
HIRF: High intensity radiated field
MLG: Multilayer graphene
MMG: Multiscale Maxwell Garnett
RAM: Radar absorbing material
RGO: Reduced GO
2D: Two-dimensional
